# Physical Therapy for Adults with Heart Failure

**DOI:** 10.1298/ptr.R0024

**Published:** 2023-03-28

**Authors:** Yi-Chen WU, Chiao-Nan CHEN

**Affiliations:** ^1^Department of Physical Therapy and Assistive Technology, School of Biomedical Science and Engineering, National Yang Ming Chiao Tung University, Taiwan

**Keywords:** CHF, COVID-19, ADHF, Rehabilitation, HF

## Abstract

Heart failure (HF) is a complex clinical syndrome caused by structural and/or functional abnormalities that results in significant disease burdens not only to the patients and their families but also to the society. Common symptoms/signs of HF include dyspnea, fatigue, and exercise intolerance, which significantly reduce the quality of life of individuals. Since the coronavirus disease 2019 (COVID-19) pandemic in 2019, it has been found that individuals with cardiovascular disease are more vulnerable to COVID-19-related cardiac sequelae including HF. In this article, we review the updated diagnosis, classifications, and interventional guidelines of HF. We also discuss the link between COVID-19 and HF. The latest evidence about physical therapy for patients with HF in both the stable chronic phase and acute cardiac decompensation phase is reviewed. Physical therapy for HF patients with circulatory support devices is also described.

**H**eart failure (HF) is a complex clinical condition caused by structural and/or functional abnormalities or cardiac injury^1)^. The diagnosis of HF requires both the presence of HF symptoms/signs and the objective evidence of cardiac dysfunction/injury. Symptoms and signs of HF include shortness of breath, fatigue, persistent coughing or wheezing, peripheral edema, lack of appetite, and elevated resting heart rate (HR). The objective diagnosis tests of HF include echocardiography, natriuretic peptide (NP), electrocardiogram (ECG), and chest X-ray (CXR)^2)^. Echocardiography is the key assessment of cardiac function that provides information on ventricular systolic function, cardiac chamber sizes, wall motion, and eccentric/concentric left ventricular hypertrophy (LVH). Plasma NPs such as N-terminal pro-B-type natriuretic peptide (NT-proBNP) and B-type natriuretic peptide (BNP) are produced from cardiac tissue when the cardiac tissue is stretched by pressure or volume overload. Thus, elevated NP (NT-proBNP ≥125 pg/mL or BNP ≥35 pg/mL) suggests increased cardiac wall stress^2,3)^. The ECG reveals abnormal cardiac electrophysiology such as arrhythmias, LVH, atrial fibrillation, and the widened QRS complex (the Q wave, the R wave, and the S wave). Last, CXR is often used to help the differential diagnosis of HF^2)^.

The etiology of HF is various, including coronary artery disease, hypertension (HTN), valvular heart disease, arrhythmias, cardiomyopathy, congenital heart disease, pericardial disease, infection, drug-induced cardiotoxicity (such as anticancer medications anthracyclines and trastuzumab), metabolic/autoimmune diseases, and neuromuscular disease^2)^. The pathophysiology of HF can be described from the structural and functional perspectives. From a structural perspective, HF is classified into left-sided or right-sided HF. Left-sided HF reduces cardiac output, increases fluid retention in the atrium, and eventually results in pulmonary congestion and edema. Right-sided HF results in systemic (venous) congestion. The dominant symptoms of left-sided HF are dyspnea, paroxysmal nocturnal dyspnea, orthopnea, and cough. The dominant symptoms of right-side HF are jugular venous distention, peripheral edema, ascites, and pleural effusion^4,5)^. From the functional perspective, HF is classified into systolic HF or diastolic HF. Systolic HF is characterized by impaired cardiac contractility, resulting in reduced cardiac output. Diastolic HF is characterized by impaired relaxation of cardiac muscles during the ventricular filling stage, which also results in reduced cardiac output^4,6)^. No matter left-sided or right-sided HF, systolic or diastolic HF, the end result is reduced cardiac output. Reduced cardiac output not only leads to hypotension, cardiogenic shock, and organ dysfunction but also results in elevated cardiac pressure that results in pulmonary congestion and poor ventilatory efficiency^7–9)^.

## HF: Classification

The types of HF are classified based on the left ventricular ejection fraction (LVEF) assessed by echocardiogram. Individuals with LVEF ≤40% or ≥50% at the first diagnosis and the follow-ups are defined as those with heart failure with reduced ejection fraction (HFrEF) and heart failure with preserved ejection fraction (HFpEF), respectively^10)^. Individuals with LVEF 41%–49% at the first diagnosis and the follow-ups are defined as heart failure with mildly reduced ejection fraction (HFmrHF). Individuals with previous LVEF ≤40% and a follow-up LVEF >40% are defined as heart failure with improved ejection fraction (HFimpEF)^10)^. HFrEF is used to be called systolic HF and HFpEF is used to be called diastolic HF. Individuals with HFrEF are usually younger than individuals with HFmrEF and HFpEF, and often occurs in individuals with a history of myocardial infarction^11)^. HFmrEF occurs more frequently in individuals with a history of diabetes mellitus, hyperlipidemia, peripheral artery disease, renal failure, dialysis, and ischemic events^11)^. HFpEF occurs more frequently in individuals with a history of HTN, depression, and cerebral vascular accidents^11)^. Compared to HFrEF, obesity and low physical activity are more associated with the risk of HFpEF^12)^. Last, compared to individuals with HFrEF, individuals with HFimpEF have a lower prevalence of ischemic heart disease, a higher prevalence of HTN, and a more favorable outcome^13)^.

**Fig. 1.**
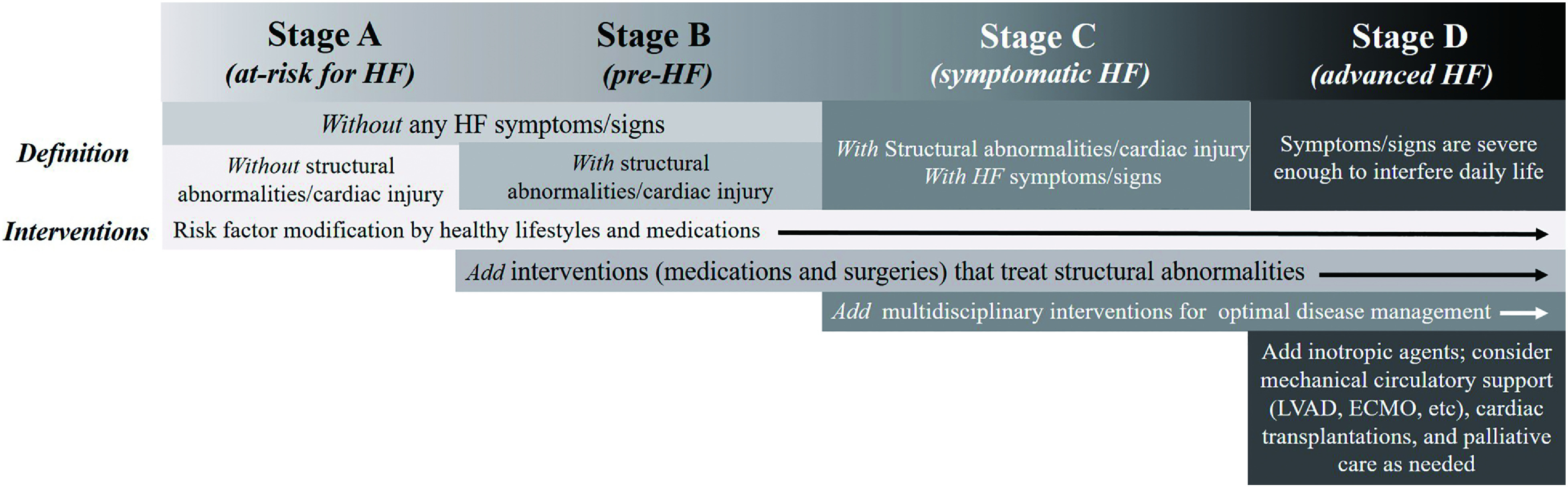
The definition and suggested interventions for patients with HF at different stages

HF, heart failure; LVAD, left ventricular assist device; ECMO, extracorporeal membrane oxygenation

The stages of HF are defined by the American College of Cardiology (ACC) and the American Heart Association (AHA) as stages A, B, C, and D. Stage A indicates individuals with risk of HF (e.g., HTN, diabetes, obesity, and atherosclerotic cardiovascular diseases) but without any HF symptoms/signs and structural abnormalities/cardiac injury. Stage B indicates individuals with structural abnormalities/cardiac injury but without HF symptoms/signs. Stage C indicates individuals with both HF symptoms/signs and structural abnormalities/cardiac injury. Stage D indicates individuals whose symptoms/signs are severe enough to interfere with daily life^10)^. The treatment aim for individuals with stage A and B is primary prevention. Individuals with stage A HF should modify risk factors via healthy lifestyles (e.g., regular exercise, avoiding obesity, and not smoking) and medications (e.g., statin, beta-blockers, and renin–angiotensin system inhibitors). For individuals with stage B, treatment should add interventions that treat structural abnormalities including surgeries (e.g., valve replacement and coronary revascularization) and implantable cardioverter-defibrillator (ICD) implantation in addition to risk modification by lifestyle and medications. The treatment aims for individuals with stage C HF is improving symptoms, preventing the worsening of symptoms, and reducing mortality and morbidity. Thus, in addition to risk modification, interventions including disease management education and support from a multidisciplinary team (e.g., vaccination, adherence to medication, restriction of dietary sodium, exercise, and cardiac rehabilitation), medications (e.g., diuretics, renin–angiotensin system inhibitors, beta-blockers, and digoxin), implantable electrical interventions (e.g., ICD and cardiac resynchronization therapy), and surgeries (e.g., valve replacement and coronary revascularization) are needed. The treatment aim for individuals with stage D HF is improving functional status, quality of life (QoL), and life span. Specialty referral for advanced care is suggested (e.g., left ventricular assist device [LVAD], cardiac transplantation, and palliative care) in addition to the suggested interventions for stages A–C HF (Fig. 1)^10,14)^.

**Fig. 2.**
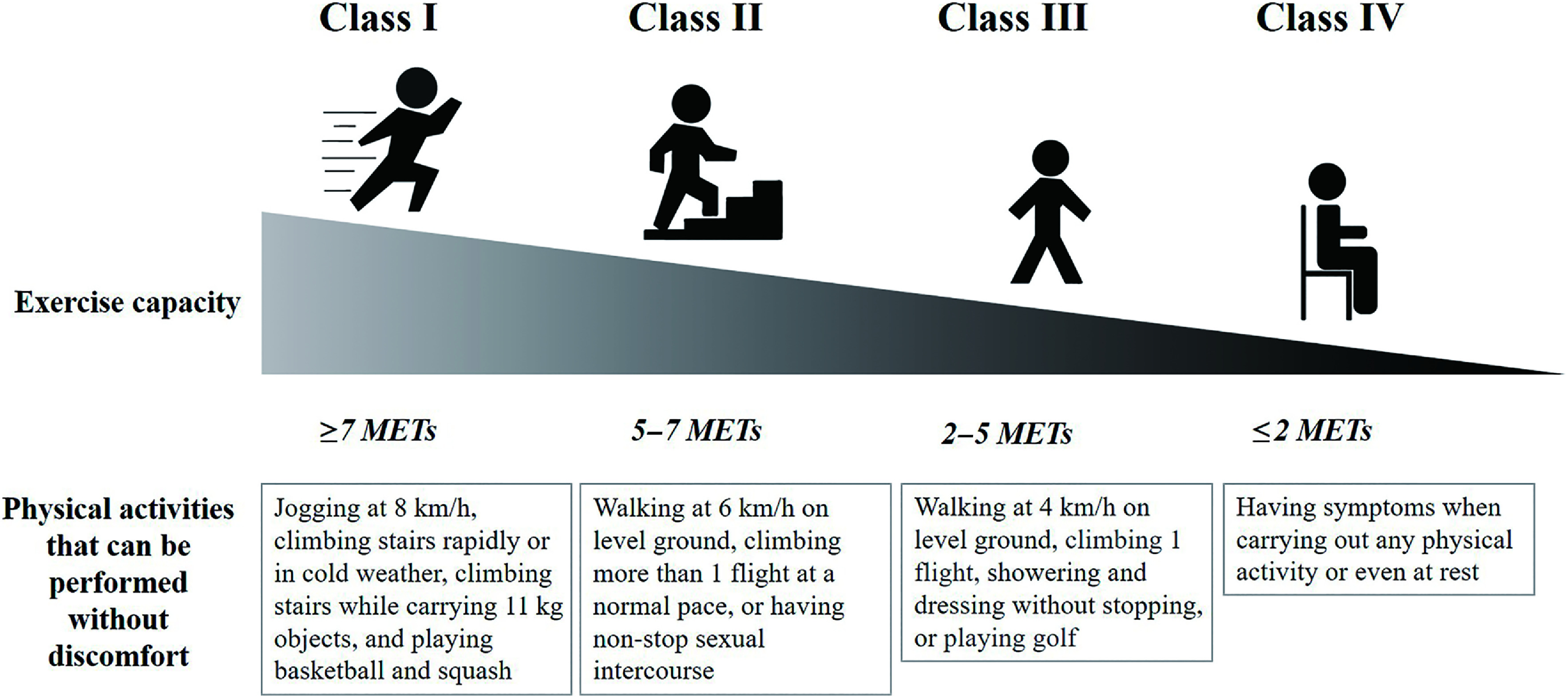
NYHA functional classification

METs, metabolic equivalents; NYHA, New York Heart Association

The functional capacity of individuals with HF is classified by the New York Heart Association (NYHA). NYHA classification is a subjective assessment and classifies HF as I, II, III, and IV. Class I refers to individuals who do not have limitations in physical activity and can perform activities requiring ≥7 metabolic equivalents (METs) such as jogging at 8 km/h, climbing stairs rapidly or in cold weather, climbing stairs while carrying 11 kg objects, and playing basketball and squash. Class II refers to individuals who have a slight limitation of physical activity. Individuals with NYHA class II can perform activities requiring 5–7 METs (such as walking at 6 km/h on level ground, climbing more than 1 flight at a normal pace, or having non-stop sexual intercourse) without discomfort, but feel fatigued, or have palpitations or dyspnea during activities that require more than 7 METs. Class III refers to individuals who have marked limitations of physical activity. Individuals with NYHA class III are comfortable at rest, can perform activities requiring 2–5 METs (such as walking at 4 km/h on level ground, climbing 1 flight, showering and dressing without stopping, or playing golf) without discomfort, but feel fatigued, or have palpitations or dyspnea during activities that require more than 5 METs. Class IV refers to individuals who have symptoms when carrying out any physical activity or even at rest (Fig. 2)^4,10)^.

Depending on clinical presentations, HF is usually divided into chronic heart failure (CHF) and acute decompensated heart failure (ADHF)^2)^. Individuals with CHF are under optimal medical therapy and are asymptomatic or only mildly symptomatic^15)^. ADHF is a new onset or worsening of HF symptoms where individuals suffer from acute congestion symptoms, including exacerbated dyspnea and fatigue, weight gain, jugular venous distention, pulmonary or peripheral edema, or cardiogenic shock. ADHF results from a gradual increase of intraventricular pressure due to acute coronary syndrome, arrhythmias, or cardiac inflammation^10,16)^. Studies found that ADHF is highly associated with high mortality and hospital readmission rates^2,5,17)^.

## Coronavirus Disease 2019 (COVID-19) and HF

Severe acute respiratory syndrome coronavirus 2 (SARS-CoV-2), also called COVID-19, has infected many people worldwide and caused many deaths since 2019^18)^. According to the data from the World Health Organization (WHO) in April 2022, more than 632 million people confirmed COVID-19 and more than 6.5 million people died from the disease^19)^. People with COVID-19 present various symptoms with different severity. Individuals with mild illness might have fever, cough, sore throat, malaise, headache, muscle pain, nausea, vomiting, diarrhea, or loss of taste and smell, but do not have dyspnea or abnormal chest imaging. Individuals with moderate illness have infections involving the lower respiratory tract and have oxygen saturation (SpO_2_) ≥94% on room air. Individuals with severe illness have resting respiratory rate >30 breaths/min or SpO_2_ <94% on room air. Individuals with critical illnesses have respiratory failure, septic shock, and/or multiple organ dysfunction^20)^. In addition to the common symptoms stated above, some individuals develop COVID-19-related cardiac impairments during the acute onset or recovery phase. COVID-19-related cardiac impairments include acute myocardial infarction^21)^, myopericarditis^22)^, myocarditis^23)^, cardiomyopathy^24)^, and HF^25)^. The reported prevalence of COVID-19-related diastolic dysfunction of cardiac muscles, decreased LVEF, pulmonary HTN, pericardial effusion, and cardiac global hypokinesis in adults is 9.9%, 2.8%, 1.8%, 1.5%, and 0.8%, respectively^26)^. A follow-up study found that 2% of adults with acute COVID-19 develop HF one year after hospital admission^27)^. The clinical manifestations of COVID-19-related cardiac impairments are chest pain, shortness of breath, and fatigue^21–25)^.

Noteworthy, individuals with cardiovascular diseases are more vulnerable to COVID-19-related cardiac sequelae and high mortality than individuals without cardiovascular diseases^28)^. A study showed that 49% of individuals who died from COVID-19 had pre-existing HF^29)^. The reason why individuals with cardiovascular diseases are more likely to have COVID-19-related cardiac sequelae is that the content and activity of angiotensin-converting enzyme 2 (ACE2), the key receptor of SARS-CoV-2, in cardiomyocytes are greater in individuals with cardiovascular diseases than in healthy individuals^30)^. The binding of SARS-CoV-2 to ACE2 activates cellular signaling pathways and causes an immune response that leads to subsequent inflammation and myocardial injury^31,32)^. Indeed, people with COVID-19-related cardiac symptoms are found to have elevated biomarkers of cardiac damage such as NT-proBNP and troponin^33–36)^. Taken together, the impact of COVID-19 infection on people varies a lot, from mild to critically ill. COVID-19 infection may affect cardiac function, especially in individuals with pre-existing cardiovascular diseases.

About 10%–20% of COVID-19 survivors develop post-COVID-19 symptoms. A meta-analysis examined the prevalence of post-COVID-19 symptoms at different follow-up periods and found that the top two symptoms at 3–6 months, 6–9 months, 9–12 months, and longer than 12 months are fatigue/dyspnea, effort intolerance/fatigue, fatigue/dyspnea, and fatigue/dyspnea, respectively^37)^. Objective measurement of physical fitness also found that individuals who recover from severe/critical COVID-19 have the lowest peak oxygen consumption (VO_2_ peak) and anaerobic threshold than individuals who recover from mild/moderate COVID-19 or individuals without COVID-19^38,39)^. Deconditioning due to immobilization and inactivity is the main cause of fatigue and exercise intolerance of COVID-19 survivors^40)^. Nevertheless, left ventricular end-diastolic volume (LVEDV), LVEF, and cardiac output at every stage of stress exercise test are found lower in 3-month follow-up of COVID-19 survivors than in control individuals^38)^. This finding suggests that cardiac sequelae might also play a role in the deceased exercise capacity of COVID-19 survivors.

## Physical Therapy for Individuals with HF

### Education

Education on disease self-management is critical for individuals with HF. Individuals who have better disease self-management (i.e., adherence to medications, proper diet and exercise, and monitoring any exacerbation of symptoms) have a better QoL and lower hospital readmission rate and mortality^41)^. All individuals with HF should be educated on the importance of adherence to medications and recognizing the signs/symptoms of exacerbation of HF. Individuals should measure body weight daily and see a doctor when body weight increases to 0.9–1.4 kg in 24 hours or 5 kg over 3 days^4)^. In terms of diet, individuals with HF are suggested to restrict dietary sodium (<2300 mg/d) and have healthy dietary patterns such as Mediterranean diet or Dietary Approaches to Stop Hypertension diet^10)^. Physical inactivity is highly associated with greater mortality in people with CHF. Low physical activity (<25,000 steps/week) is a predictor of mortality for HF patients with NYHA classes II–III^42,43)^. Conversely, regular exercise improves exercise capacity and QoL and reduces mortality in individuals with CHF^44)^. Taken together, good disease self-management is critical for individuals with HF. In addition to stressing the importance of exercise, physical therapists should also remind individuals of other components of disease self-management.

### Exercise prescription for individuals with ADHF

Physical function such as sit-to-stand and walking ability is severely impaired in older individuals with ADHF. They are also often frail, depressed, and with impaired cognition^45)^. While most exercise training studies exclude individuals with ADHF, a recent Rehabilitation Therapy in Older Acute Heart Failure Patients (REHAB-HF) study provided evidence on the beneficial effects of an early and progressive rehabilitation intervention on the physical function of older adults who are hospitalized for ADHF. REHAB-HF study found that 3 months of physical rehabilitation initiated during hospitalization improved physical performance, endurance, and QoL of older individuals hospitalized for ADHF^46)^. The aim of REHAB-HF is to enhance physical function of individuals with ADHF so that they can be independent in the activities of daily living^47)^. Due to fragility and poor functional status of individuals, a supervised and comprehensive exercise program is suggested^45)^. For all individuals, the intervention includes balance, mobility, strength, and endurance training where the proportion of each type of exercise is different based on the functional level of individuals. While the strength training accounts for about 1/3 of the overall exercise time for all individuals, a greater proportion of training time shifts from balance and mobility training toward endurance training when individuals have better functional levels. Regarding intensity, the training begins with low intensity and high frequency, and progresses to high intensity with low frequency. Specifically, during hospitalization, the intervention is 45 minutes daily where individuals are allowed to rest when they need; the target intensity is at rating of perceived exertion (RPE) ≤12^46)^. After discharge, individuals attend outpatient rehabilitation 3 times per week, 60 minutes each time with target RPE 13 (somewhat hard) for endurance training and RPE 15–16 (hard) for strength training^47)^. Detailed exercise prescription for different functional levels of individuals in the REHAB-HF study is shown in Table 1^46,48)^.

**Table 1.** Comprehensive exercises for individuals with ADHF

Functional level of patients	Level 1 Gait speed ≤0.4 m/s Unable to stand with feet together unsupported Unable to stand from sitting position without hand support Can walk <2 minutes (with/ without the assistive device)	Level 2 Gait speed 0.4–0.6 m/s Can stand with feet together for 10 seconds Can stand from sitting position without hand support at least one time Can walk 2–10 minutes (with/ without the assistive device)	Level 3 Gait speed 0.6–0.8 m/s Can stand unsupported and reach forward 25.4 cm Complete 5 times sit to stand in 15–60 seconds Can walk 10–20 minutes (with/without the assistive device)	Level 4 Gait speed >0.8 m/s Can perform single-leg standing for 10 seconds Complete 5 times sit to stand ≤15 seconds Can walk ≥20 mins (with/ without the assistive device)
Mobility training	Start with slow speed and then add start/stop walking events, change directions during walking			
Balance training	Static balance: maintain standing with wider base of support Dynamic balance: reach forward and backward	Static balance: maintain standing with feet together, semi-tandem standing, tandem standing Dynamic balance: reaching forward beyond the base of support	Integrate balance training with endurance training such as walking with turning and stopping abruptly, dual task	
Strength training	Focuses on functional strengthening exercises such as sit-to-stand, calf raising, and step-up			
Endurance training	Start with repeated bouts of ambulation with rest periods as needed. Initial duration is 5–10 minutes and gradually progresses to 40 minutes.			
ADHF, acute decompensated heart failure				

### Exercise prescription for individuals with CHF

It is evident that exercise improves functional capacity and health-related QoL and reduces hospital readmissions for individuals with CHF. The American Physical Therapy Association (APTA) published a physical therapist clinical practice guideline for the management of individuals with HF in 2020 where decision flowcharts, exercise prescriptions, and the levels of evidence are well described^4)^. In general, individuals without signs/symptoms of acute cardiac decompensation and uncontrolled diseases are safe to receive exercise training. Aerobic exercise (both continuous and high-intensity interval), resistance exercise, and inspiratory muscle training are recommended for individuals with CHF^4)^. The exercise duration per session is 30–60 minutes. Exercise frequency is 3–7 days/week for aerobic exercise and inspiratory muscle training and 2–3 days/week for resistance exercise^49–55)^. The intensity of aerobic exercise in the form of moderate-intensity continuous training (MICT) is 40%–70% VO_2_ peak/HR peak/peak work rate or RPE 11–15^49,50)^. For aerobic exercise in the form of high-intensity-interval exercise training (HIIT), patients are asked to complete 2–6 cycles of 3–4 minutes of high intensity (75%–80% heart rate reserve [HRR] or 70%–95% VO_2_ peak/HR peak) with 2–3 minutes of low-intensity (40%–50% HRR or 50%–70% VO_2_ peak/HR peak) exercise^51,52)^. The intensity of resistance exercise begins with 40%–50% one repetition maximum (1RM) and then progressively increases to 60%–85% 1RM. Resistance exercise training usually includes 8–10 movements that involve major muscle groups. Each movement repeats 8–15 times for 2–3 sets^53,54)^. The intensity of inspiratory muscle training is 30%–60% maximal inspiratory pressure^55)^.

Regarding the level of monitoring during exercise, it is suggested to monitor ECG and blood pressure (BP) at the beginning sessions of training for individuals with NYHA I/II until they know how to monitor signs/symptoms by themselves. For individuals with NYHA III/IV, individuals having an systolic BP drops below resting levels during the exercise test, and individuals having angina or ischemic ST depression in the ECG at exercise intensity <6 METs, more than 12 sessions of continuous ECG and BP monitoring is suggested until the safety is established^56)^. Regarding the training effects of exercise, a meta-analysis found that 8–12 weeks of both MICT and HIIT improve exercise capacity and QoL of individuals with HFrEF^ 49–52)^. The average improvement of VO_2_ peak is 0.69–2.08 mL/kg/min; the average improvement in the six-minute walking distance test (6MWD) is 21–25.67 m; the average improvement in Minnesota Living with Heart Failure Questionnaire (MLHFQ) is 5.9–10.86^49–52)^. Only one recent randomized clinical trial compared the effects of MICT (40 minutes/session; 5 sessions/week) and HIIT (38 minutes/session; 3 sessions/week) on individuals with HFpEF ^57)^. It found that the improvement of VO_2_ peak and QoL after training was similar between MICT and HIIT (1.6 mL/kg/min vs. 1.1 mL/kg/min)^57)^. It is important to note that while the effects of MICT and HIIT on exercise capacity are similar, HIIT seems to be more effective in heart remodeling^50–54)^. Studies show that 8–24 weeks of HIIT, but not MICT, increases LVEF (1.32%) and LVEDV of patients with HFrEF^51,52)^. Recent meta-analysis showed that 8–20 weeks of resistance training improves cardiac performance, muscle strength, exercise capacity, and QoL of individuals with HFrEF^53,54)^. The average improvement of VO_2_ peak is 2.49 mL/kg/min, the average improvement in the 6MWD is 49.94 meters, and the average improvement in MLHFQ is 8.25^53,54)^. Last, daily inspiratory muscle training improves inspiratory muscle strength, exercise capacity, and QoL of individuals with HFrEF^55)^. The average improvement of VO_2_ peak is 3.75 mL/kg/min and the average improvement in the 6MWD is 81.2 m^55)^. One point worth noting is that most studies to date focus on individuals with HFrEF or have mixed types of HF as study subjects. Since different types of HF have their unique pathology and the characteristics of patients with different types of HF are different, further research is needed to explore the possible type-specific effects of exercise training.

### Physical therapy for HF patients with mechanical devices

The most common implantable devices seen in individuals with HF are ICDs and cardiac resynchronization devices (pacemakers). An ICD is often implanted in patients who have uncontrolled arrhythmia (such as ventricular tachycardia or fibrillation). An ICD delivers an electrical shock to reset the heartbeat when individuals have a life-threatening arrhythmia. A pacemaker is a cardiac resynchronization device and is often implanted in patients whose ventricles could not contract coordinately. A pacemaker delivers electrical signals to trigger the coordinated ventricular contractions, thus correcting irregular or slow heartbeats. Supervised exercise training including aerobic and resistance exercise is safe and effective for individuals with ICD and pacemakers. An exercise test is recommended to evaluate the risk of HR reaching the ICD intervention zone^58)^. The AHA suggests that the target HR of exercise is set at 10–15 beats/min lower than the ICD therapy rate threshold. The common exercise prescription for individuals with ICD and/or pacemakers is 3–5 days/week, 30–90 minutes/session, and with intensity 50%–80% VO_2_ peak/80%–85% HRR^58)^. More than 3 months of HR or RPE monitoring during exercise is necessary^58,59)^. It is found that ICD discharges during exercise are very rare (0.9%) with appropriate exercise prescription and monitoring^58)^. Meta-analysis that examined the effects of supervised exercise training on HF patients with ICD and/or pacemakers showed that 8–12 weeks of both MICT and HIIT improve exercise capacity and QoL of patients^58,60)^. Importantly, individuals who receive exercise training have 7% less ICD shocks compared to individuals who do not receive exercise training^58)^. Collectively, HF patients with ICDs and/or pacemakers are safe to receive exercise training. Exercise improves the exercise capacity and QoL of this population.

For individuals with ADHF and cardiogenic shock (i.e., hypotension accompanied by hypoperfusion of organs), mechanical circulatory support devices are commonly used. Intra-aortic balloon pump is a device that helps reduce the afterload of the heart and increase cardiac perfusion, which has been the most widely used device since the 1960s^61)^. Recently, the use of ventricular assist devices (VADs) and extracorporeal membrane oxygenation (ECMO) has dramatically increased because they provide a greater level of hemodynamic support. VAD is a device that helps the pumping of the heart, thus reducing the workload of the heart. ECMO is the device that replaces the functions of the heart and lung, thus decreasing the workload of them. For critically ill individuals, joint contracture, muscle atrophy, and deconditioning are the common sequelae of bedridden. Early mobilization, including ambulation, is a safe and feasible intervention that benefits individuals’ functional outcomes^62)^. A multidisciplinary team must include physicians, nurses, respiratory therapists, physical therapists, occupational therapists, and perfusionists to provide comprehensive care for critically ill individuals. Once an individual is assessed as suitable for activity by the medical team, physical therapists assess the functional level of patients and initiate early mobilization intervention, which usually requires assistance from other clinicians (such as a nurse, an occupational therapist, or a perfusionist)^62,63)^. Early mobilization exercises include range of motion exercises, resistance exercises, transfer exercises, standing, stepping, bed cycling, and ambulation where the selection of exercises is individualized based on the consciousness level, functional level, and disease severity of individuals^62,64)^. Vital signs, RPE, and circuit integrity should be monitored during the intervention. Exercise intervention is terminated when patients have hemodynamic instability, hypoxemia, dizziness, weakness, chest pain, or dyspnea^62)^. It is worth noting that for individuals with femoral access to mechanical circulatory support devices, movements that require hip flexion over 30 degrees or overextend the leg should be avoided to decrease the risk of malpositioning^65)^. Kerrigan et al. reported that 6 weeks of aerobic training (walking/cycling at 60%–80% HRR, 30 minutes/session, 3 sessions/week) improves cardiorespiratory fitness, muscle strength, and QoL of individuals who receive continuous flow LVAD implantation within 6 months^66)^. Hayes et al. reported that exercise training that includes moderate-intensity aerobic exercise and resistance training (2 sets of 10 repetitions) is feasible and safe for patients with LVAD who can walk independently. Additionally, the combined aerobic and resistance training had a trend to greater improve exercise capacity and QoL of HF patients than mobilization training (walking at moderate intensity) alone^67)^. In conclusion, HF patients with circulatory support devices can still receive physical therapy safely and effectively although equipped with circulatory support devices suggests that the patient has a significantly impaired cardiac function. Appropriate physical therapy interventions improve the physical fitness, mobility ability, and QoL of HF patients with circulatory support devices.

## Conclusions

The reduced cardiac output and poor ventilatory efficiency impair exercise tolerance of individuals with HF. The current evidence supports the safety and effectiveness of self-management and individualized exercise programs for individuals with HF. With proper exercise prescription and monitoring, patients with CHF, ADHF, or circulatory support devices can get benefit from physical therapy intervention. Worth noting, since the COVID-19 pandemic in 2019, it has been found that individuals with cardiovascular diseases are more vulnerable to COVID-19-related cardiac sequelae including HF. Therefore, physical therapists should be aware of the effects of COVID-19 on the cardiac function when treating patients with COVID-19 and pre-existing cardiovascular diseases.

## Acknowledgments

This study was supported by Yen Tjing Ling Medical Foundation (CI-110-20) and the Ministry of Science and Technology, Taiwan (MOST-109-2314-B-010-036-MY3).

## Conflict of Interest

The authors declare no conflict of interest.
